# Dihydrohomoplantagin and Homoplantaginin, Major Flavonoid Glycosides from *Salvia plebeia* R. Br. Inhibit oxLDL-Induced Endothelial Cell Injury and Restrict Atherosclerosis via Activating Nrf2 Anti-Oxidation Signal Pathway

**DOI:** 10.3390/molecules27061990

**Published:** 2022-03-19

**Authors:** Ning Meng, Kai Chen, Yanhong Wang, Jiarong Hou, Wenhui Chu, Shan Xie, Fengying Yang, Chunhui Sun

**Affiliations:** 1 School of Biological Science and Technology, University of Jinan, Jinan 250022, China; mls_mengn@ujn.edu.cn (N.M.); wangyanhong1203@163.com (Y.W.); jiaronghou@163.com (J.H.); cwh15254138019@163.com (W.C.); xieshan2022@163.com (S.X.); 2 New Drug Evaluation Center, Shandong Academy of Pharmaceutical Sciences, Jinan 250101, China; chenkai@sdaps.cn; 3 Institute for Advanced Interdisciplinary Research (iAIR), University of Jinan, Jinan 250022, China

**Keywords:** homoplantaginin, atherosclerosis, vascular endothelial cell, oxidized low-density lipoprotein, Nrf2

## Abstract

Oxidized low-density lipoprotein (oxLDL)-induced endothelium injury promotes the development of atherosclerosis. It has been reported that homoplantaginin, a flavonoid glycoside from the traditional Chinese medicine *Salvia plebeia* R. Br., protected vascular endothelial cells by inhibiting inflammation. However, it is undetermined whether homoplantaginin affects atherosclerosis. In this study, we evaluated the effect of homoplantaginin and its derivative dihydrohomoplantagin on oxLDL-induced endothelial cell injury and atherosclerosis in apoE-/- mice. Our results showedthat both dihydrohomoplantagin and homoplantaginin inhibited apoptosis and the increased level of ICAM-1 and VCAM-1 in oxLDL-stimulated HUVECs and the plaque endothelium of apoE-/- mice. Additionally, both of them restricted atherosclerosis development of apoE-/- mice. Mechanistic studies showed that oxLDL-induced the increase in ROS production, phosphorylation of ERK and nuclear translocation of NF-κB in HUVECs was significantly inhibited by the compounds. Meanwhile, these two compounds promoted Nrf2 nuclear translocation and increased the anti-oxidation downstream HO-1 protein level in HUVECs and plaque endothelium. Notably, knockdown of Nrf2 by siRNA abolished the cell protective effects of compounds and antagonized the inhibition effects of them on ROS production and NF-κB activation in oxLDL-stimulated HUVECs. Collectively, dihydrohomoplantagin and homoplantaginin protected VECs by activating Nrf2 and thus inhibited atherosclerosis in apoE-/- mice.

## 1. Introduction

Atherosclerosis, a chronic inflammatory vascular disease, is a major cause of morbidity and mortality in the modern society. Currently, the prevention and treatment of atherosclerosis are all important areas of medical research. Although the pathological features of atherosclerosis are complex, injury of the endothelial lining of lesion-prone areas from the arterial vasculature is one of the main pathological processes of atherosclerosis [1]. Accumulating evidence has also demonstrated that oxidized low-density lipoprotein (oxLDL), an important dangerous factor in the initiation and progression of atherosclerosis, induces endothelial cell injury. In the initial stage of atherosclerosis, oxLDL activates endothelial cells by inducing the expression of several cell surface adhesion molecules including ICAM-1 and VCAM-1 which recruit circulating monocytes from the blood into intima, where monocytes differentiate into macrophages and become foam cells by internalizing modified lipoproteins [2,3]. Additionally, oxLDL could induce endothelial cell apoptosis, which results in increased vascular permeability to cells, promoting lipids deposition and the formation of necrotic core, thus contributing to the instability and rupture of atherosclerotic lesions. Therefore, attenuating oxLDL-induced endothelial cell injury may be a promising strategy to prevent the development of atherosclerosis [4].

In recent years, the importance of traditional Chinese herbal medicine in the treatment of cardiovascular diseases has attracted extensive attention from scientists [5,6]. Homoplantaginin was identified to be the main flavonoid component of traditional Chinese herbal medicine *Salvia plebeia* R. BR. (Labiatae), used for the treatment of a variety of diseases including hepatitis, cough, diarrhea, gonorrhea, menorrhagia, tumors, and hemorrhoids [7,8]. More and more research has revealed the biological activity of homoplantaginin by using of various cell and mice models [9,10,11,12]. It is reported that homoplantaginin exhibited protective effects on H_2_O_2_-injured hepatocyte cells and lipopolysaccharide-induced inflammatory liver injury [9]. In addition, homoplantaginin antagonizes oxLDL-induced foam cell formation in murine macrophages [10]. Attractively, homoplantaginin could ameliorate free fatty acid-induced endothelial insulin resistance and endothelial inflammation, suggesting homoplantaginin may be a potential candidate compound for the prevention and treatment of vascular diseases [11,12]. However, whether homoplantaginin could abolish oxLDL-induced endothelial cell injury and inhibit atherosclerosis needs further research. Therefore, in this study, we investigated the protection role and molecular mechanisms of homoplantaginin and its derivative dihydrohomoplantagin in the process of atherosclerosis, using a model of endothelial cell injury induced by oxLDL in vitro and apoE-/- atherosclerotic mice model in vivo.

## 2. Results

### 2.1. Dihydrohomoplantagin and Homoplantaginin Inhibited oxLDL-Induced Apoptosis and the Increase in ICAM-1 and VCAM-1 Protein Levels in HUVECs

**Figure 1:**
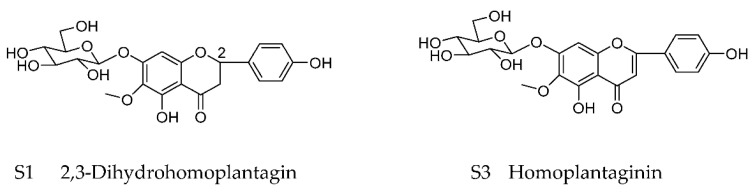
Chemical structures of dihydrohomoplantagin and homoplantaginin (named compound S1 and compound S3, respectively).

**Figure 2:**
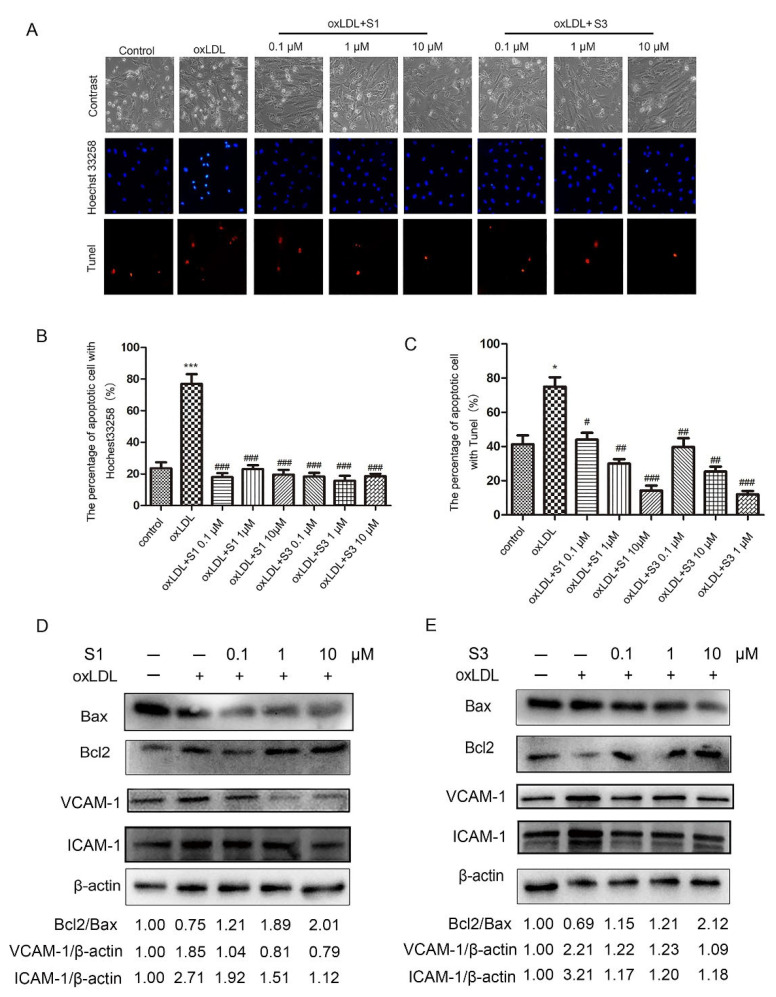
Effect of compounds S1 and S3 on oxLDL-induced apoptosis and the increase in ICAM-1 and VCAM-1 protein level in HUVECs. HUVECs were treated with oxLDL (50 μg/mL), oxLDL and indicated compounds for 6 h. (**A**) Cell apoptosis was determined by Hoechst 33258 and TUNEL staining (100×). (**B**,**C**) Quantification of percentage of HUVEC apoptosis according to panel (**A**). Data were analyzed using one-way ANOVA with Bonferroni correction. * *p* < 0.05, *** *p* < 0.001, vs. control, # *p* < 0.05, ## *p* < 0.01, ### *p* < 0.001 vs. oxLDL, *n* = 3. (**D**,**E**) Representative Western blot analysis of the Bax, Bcl-2, VCAM-1 and ICAM-1 protein levels of 3 independent experiments. Densitometry analysis of the representative images performed with ImageJ (NIH) were shown at the bottom, the untreated groups were normalized to 1.

The chemical structures of dihydrohomoplantagin and homoplantaginin (named compound S1 and compound 3, respectively) are shown in Figure 1. To elucidate the effect of S1 and S3 on vascular endothelial cell (VEC) injury under proatherogenic conditions, we first performed in vitro experiments in HUVECs with oxLDL treatment. In oxLDL-treated groups, about 80% HUVECs displayed typical characteristics of apoptosis including nuclear condensation and fragmentation using Hoechst 33258 staining, while after S1 or S3 treatment, the number of apoptotic cells significantly decreased (Figure 1). Results from TUNEL assay demonstrated that S1 and S3 significantly reduced the number of TUNEL-positive cells, respectively (Figure 2A,C). Moreover, the decreased protein level of Bcl2/Bax, a hallmark of apoptosis, was observed in oxLDL-treated HUVECs, the effects of which were reversed by S1 or S3 (Figure 2D,E).

OxLDL exposure enhanced intercellular adhesion molecule-1 (ICAM-1) and VCAM-1 expression on the endothelium, which is responsible for the oxLDL-induced endothelial injury. Therefore, we further explored the effect of dihydrohomoplantagin and homoplantaginin on the expression of ICAM-1 and VCAM-1. The results of Western blot revealed that S1 or S3 decreased oxLDL-induced up-regulation of ICAM-1 and VCAM-1 protein level in HUVECs, respectively (Figure 2D,E).

### 2.2. Dihydrohomoplantagin and Homoplantaginin Inhibited Plaque Endothelium Apoptosis and the Adhesion Factor Protein Level in apoE-/- Mice

**Figure 3:**
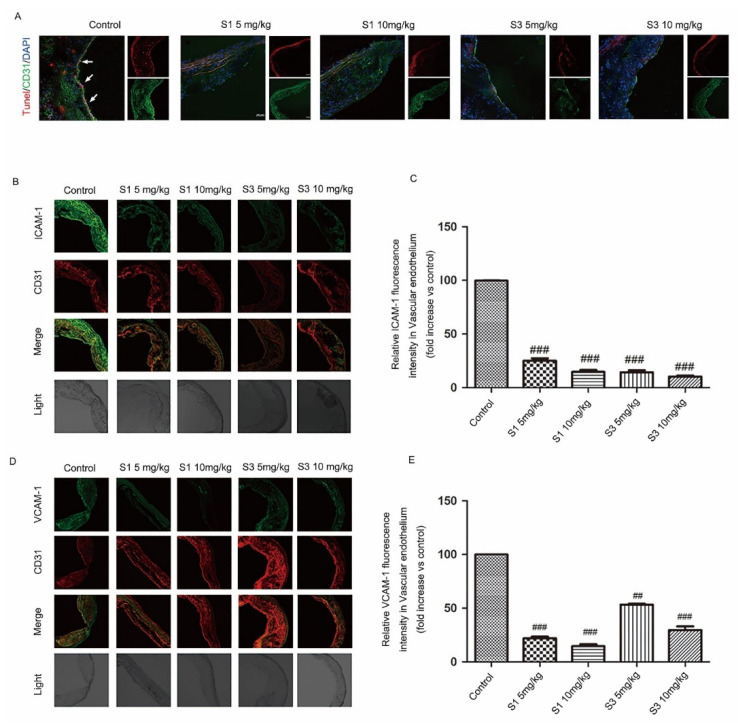
Compounds S1 and S3 inhibited endothelium apoptosis and the protein level of ICAM-1 and VCAM-1 in apoE-/- mice. (**A**) Sections from aortic roots of apoE-/- mice were labeled by TUNEL to detect apoptotic cells in endothelium. Endothelial cells were stained with CD31 (red). Nuclei (blue) were stained with DAPI (200×). Arrow indicated the apoptotic endothelial cells. A total of 6 mice per group and 3 slides per mouse were analyzed. (**B**,**D**) Immunofluorescent staining of ICAM-1 and VCAM-1 in the endothelium of aortic sinus plaque of apoE-/- mice (200×). (**C**,**E**) Quantification of ICAM-1 and VCAM-1 protein level in endothelium of 6 mice per group and 3 slides per mouse. Data were analyzed by an unpaired two-sided Student’s *t*-test. ## *p* < 0.01, ### *p* < 0.001 vs. control.

To further investigate the effect of dihydrohomoplantagin and homoplantaginin on VEC injury under proatherogenic conditions in vivo, 6 weeks of male apoE-/- mice were fed with high-fat diet for 12 weeks, and were injected intraperitoneally with compound S1 or S3 daily for 8 weeks. The body weight of mice was measured every week during compounds injection and there was no significant difference between control and compounds-treated groups (Supplemental Figure S1). Moreover, compounds S1 and S3 also had no effect on organ weights (Supplemental Table S1), suggesting they had no obvious toxicity to mice. Then, we assessed cell apoptosis by TUNEL staining (Figure 3A) and the protein level of ICAM-1 and VCAM-1 by immunofluorescence analysis of plaque endothelium from apoE-/- mice. It is observed that, in comparison with the control group, the endothelium of apoE-/- mice with S1 or S3 treatment showed less TUNEL positive area and decreased ICAM-1 and VCAM-1 protein level, suggesting S1 and S3 inhibited endothelium injury during atherosclerosis development (Figure 3B–E).

### 2.3. Dihydrohomoplantagin and Homoplantaginin Restricted Atherosclerosis Development in apoE-/- Mice

**Figure 4:**
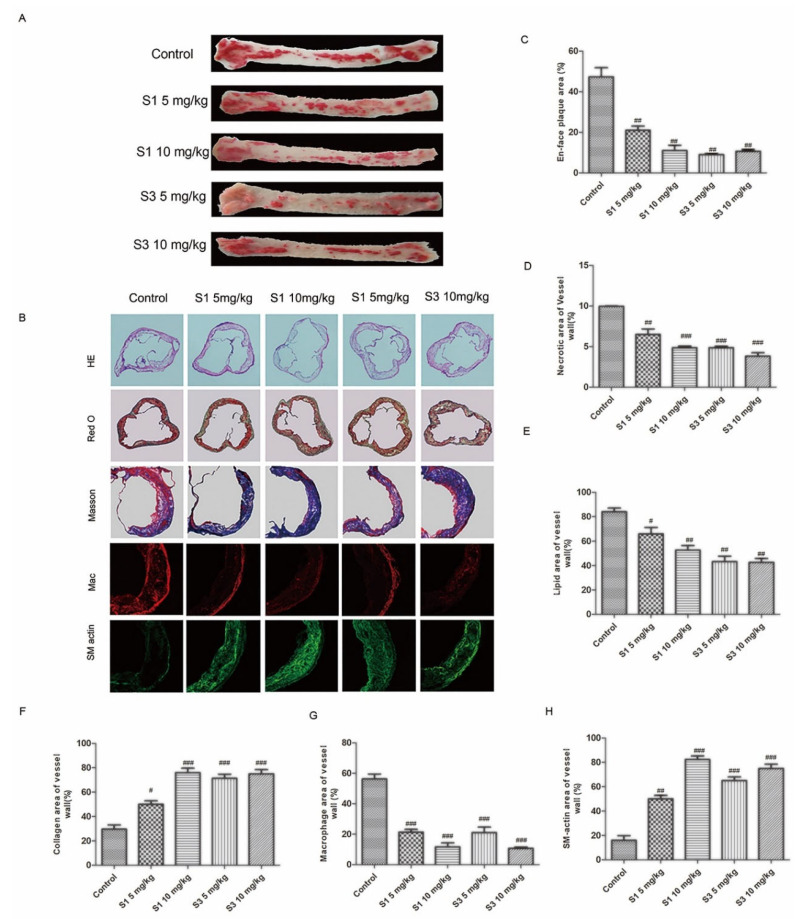
Effect of compounds S1 and S3 on the phenotype of aortic atherosclerotic plaque in apoE-/- mice. (**A**) Oil-red O staining of whole aortas of apoE-/- mice. (**B**) From the top to bottom panel, H&E staining (100×), Oil-red O staining (100×), Masson staining of frozen sections for aortic roots (200×), immunofluorescence staining for mouse a-smooth muscle with actin, macrophage with CD11C (200×). (**C**) Quantification of plaque area according to panel (**A**). (**D**) Quantification of necrotic area according to H&E staining. (**E**) Quantification of lipid area according to Oil-red O staining (**F**) Quantification of collagen area according to Masson staining. (**G**) Quantification of smooth muscle area. (**H**) Quantification of macrophage area. Data were analyzed by an unpaired two-sided Student’s *t*-test. # *p* < 0.05, ## *p* < 0.01, ### *p* < 0.001 vs. control, *n* = 6 mice per group.

Because it is believed that the injury of endothelial cell mediate the atherosclerosis progression, we further determined the anti-atherosclerotic activity of dihydrohomoplantagin or homoplantaginin in apoE-/- mice. Oil-red O staining of whole aortas and H&E staining of aortic roots demonstrated less atherosclerotic plaque areas and necrotic areas with S1 or S3 than control groups (Figure 4A,C). Moreover, these two compounds promoted the stability of plaque, displaying with the decreased lipid deposition, increased collagen content, reduced macrophage cell area and increased the number of SMCs (Figure 4B). The results of vulnerability index also demonstrated dihydrohomoplantagin or homoplantaginin enhanced atherosclerotic plaque stability (supplemental Figure S2)

### 2.4. Dihydrohomoplantagin and Homoplantaginin Inhibited ROS/ERK/NF-ĸB Signaling Pathway

**Figure 5:**
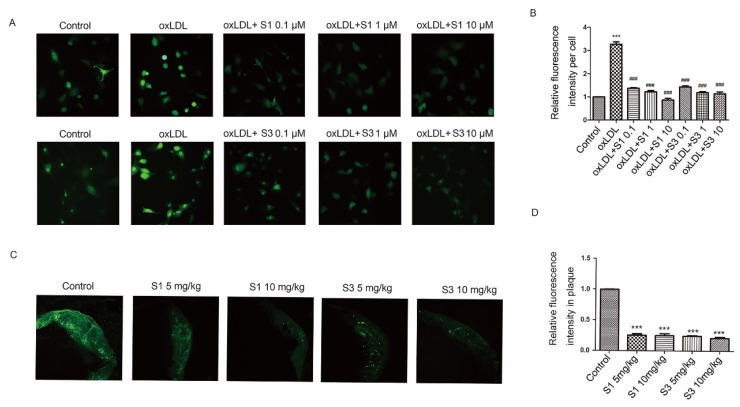
Effects of compounds S1 and S3 on the increased ROS level in oxLDL-stimulated HUVECs and plaque of apoE-/- mice. (**A**) HUVECs were treated with oxLDL (50 μg/mL), oxLDL and indicated compounds for 6 h. The ROS level in HUVEC was determined by DCHF probes (200×). (**B**) The quantification of ROS level in different group. Data were analyzed using one-way ANOVA with Bonferroni correction. *** *p* < 0.001 vs. control, ### *p* < 0.001 vs. oxLDL, *n* = 3. (**C**) Sections of aortic roots from different apoE-/- mice group were incubated with DCHF probe to detect ROS level of plaque. (**D**) The quantification of ROS level in plaque. Data were analyzed by an unpaired two-sided Student’s *t*-test. *** *p* < 0.001 vs. control, *n* = 6 mice per group.

Studies have provided compelling evidence that oxLDL stimulate excessive or sustained ROS production, which leads to vascular oxidative stress injury and thus promotes atherosclerosis [13]. Therefore, we determine whether the anti-atherosclerotic activity of dihydrohomoplantagin and homoplantaginin could be attributed to the anti-oxidative actions of these two compounds. The results revealed that incubation of HUVECs with oxLDL increased the cellular ROS level and this was strongly suppressed by S1 or S3 (Figure 5A,B). Moreover, these two compounds significantly inhibited the ROS level of atherosclerotic lesion in apoE-/- mice, respectively (Figure 5C,D).

**Figure 6:**
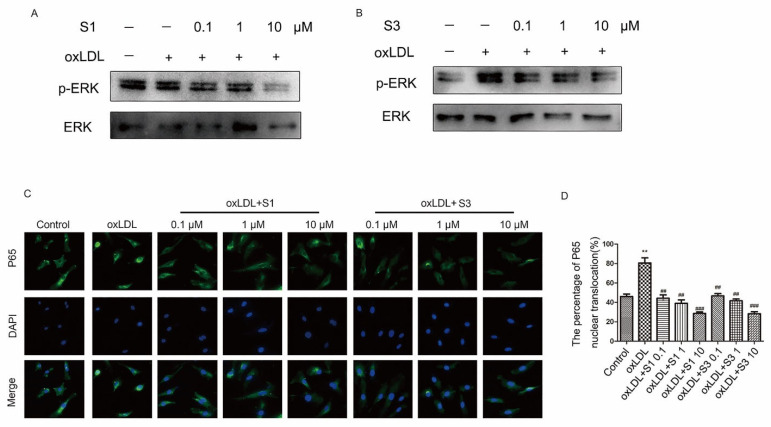
Effect of compounds S1 and S3 on the phosphorylation of ERK and NF-κB nuclear translocation in oxLDL-treated HUVECs. HUVECs were treated with oxLDL (50 μg/mL), oxLDL and indicated compounds for 6 h. (**A**,**B**) Representative Western blot analysis of pERK and ERK of 3 independent experiments.(**C**) Fluorescent micrographs showed the distribution of NF-κB p65 in HUVECs (200×). (**D**) Quantification of NF-κB p65 nuclear translocation. Data were analyzed using one-way ANOVA with Bonferroni correction. ** *p* < 0.01 vs. control, ## *p* < 0.01, ### *p* < 0.001 vs. oxLDL, *n* = 3.

Research from another group has shown that the increased ROS level could activate ERK/NF-κB signaling pathway, and finally promote the up-regulation of adhesion molecule expression in vascular endothelial cells [14]. Here, we further investigated the effect of dihydrohomoplantagin and homoplantaginin on ERK/NF-κB activation. Consistent with other studies, the phosphorylation of ERK increased and NF-κB translocated into the nuclei in oxLDL-treated HUVECs, and these were dramatically inhibited by S1 or S3 (Figure 6).

### 2.5. Dihydrohomoplantagin and Homoplantaginin Activated Nrf2 /HO-1 Anti-Oxidation Signal Pathway

**Figure 7:**
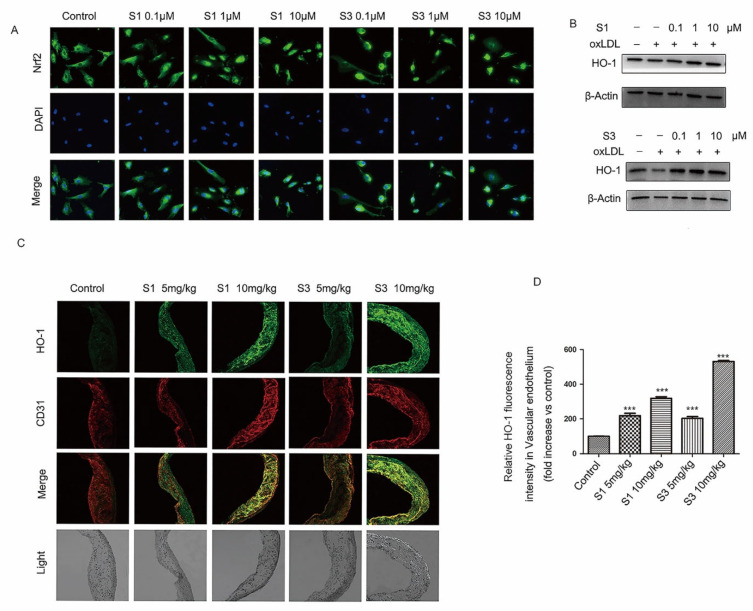
Effect of compounds S1 and S3 on Nrf2 translocation and the target gene HO-1 expression in HUVECs and endothelium of apoE-/- mice. (**A**) HUVECs were incubated with indicated compound for 6 h, the Nrf2 distribution in HUVECs were determined by Immunofluorescence staining, *n* = 3. (**B**) HUVECs were treated with oxLDL (50 μg/mL), oxLDL and indicated compounds for 6 h. Representative Western blot analysis of HO-1 of 3 independent experiments. (**C**) Double-stained images of colocalization (yellow) of HO-1 with CD31-positive cells in plaque endothelium from apoE-/- mice (200×). (**D**) Quantification of HO-1 protein level in endothelium. Data were analyzed by an unpaired two-sided Student’s *t*-test. *** *p* < 0.001, vs. control, *n* = 6 mice per group.

Nuclear factor erythroid-related factor 2 (Nrf2) is well demonstrated to play a central role in the protection of cells against oxidative damage. When Nrf2 is activated, it quickly translocates into the nucleus and increases the expression of antioxidant genes, such as heme oxygenase 1 (HO-1). To clarify the mechanisms of dihydrohomoplantagin and homoplantaginin anti-oxidation, we examined whether dihydrohomoplantagin and homoplantaginin affect Nrf2 /HO-1 anti-oxidation signal pathway. Immunofluorescence staining results showed that both compounds increased nuclear Nrf2 protein level compared with control groups (Figure 7A). Moreover, the protein levels of HO-1 were enhanced by compounds in oxLDL-stimulated HUVECs and plaque endothelium of apoE-/- mice (Figure 7B–D). These results suggested dihydrohomoplantagin and homoplantaginin activated Nrf2 /HO-1 anti-oxidation signal pathway.

### 2.6. Dihydrohomoplantagin and Homoplantaginin Inhibition of oxLDL-Induced VEC Injury and ROS/NF-κB Activation Were Nrf2-Dependent

**Figure 8:**
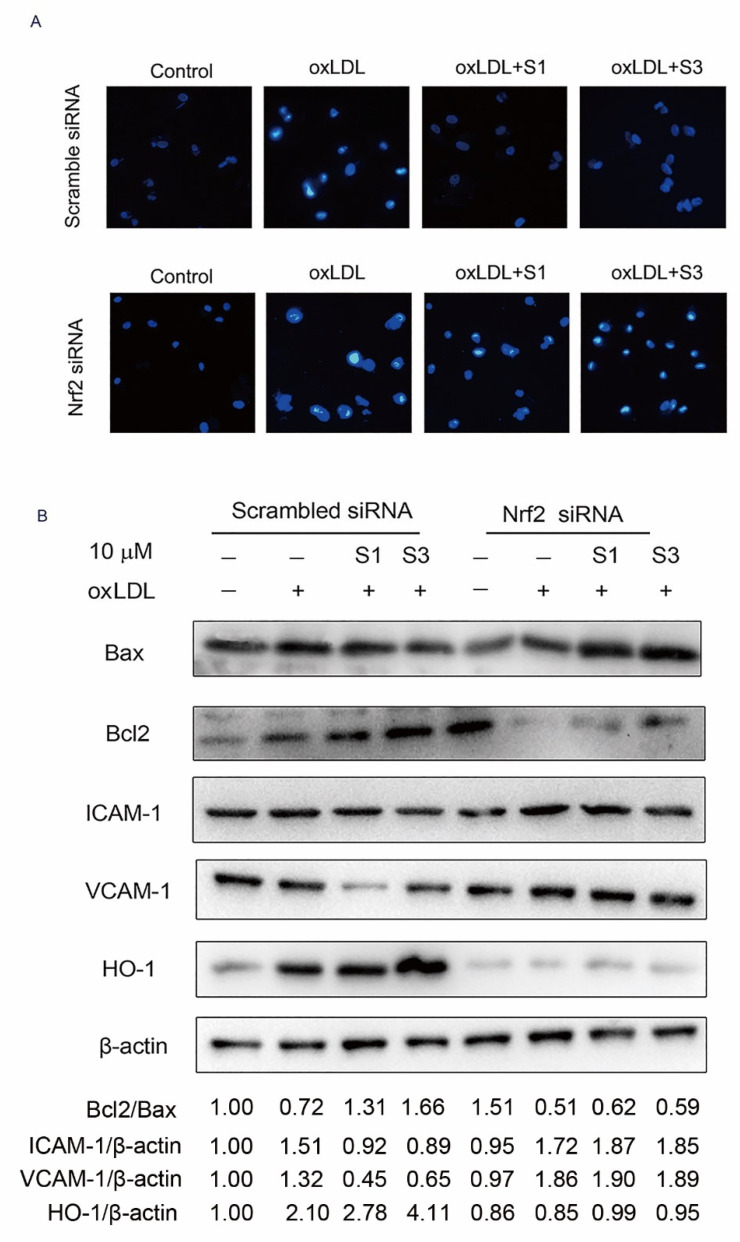
Effects of compounds S1 and S3 on cell apoptosis and protein level of Bcl2/Bax, ICAM-1, VCAM-1 and HO-1 in Nrf2-knockdown HUVECs. HUVECs were treated with 40 nM scramble siRNA or Nrf2 siRNA for 48 h and treated with oxLDL (50 μg/mL) or oxLDL and indicated compound for up to 6 h. (**A**) Cell apoptosis was determined by Hoechst 33258 (100×), *n* = 3. (**B**) Representative Western blot analysis of Bcl2, Bax, ICAM-1, VCAM-1 and HO-1 protein expression of 3 independent experiments. Densitometry analysis of the representative images performed with ImageJ (NIH) were shown at the bottom, the untreated groups in Scramble siRNA were normalized to 1.

**Figure 9:**
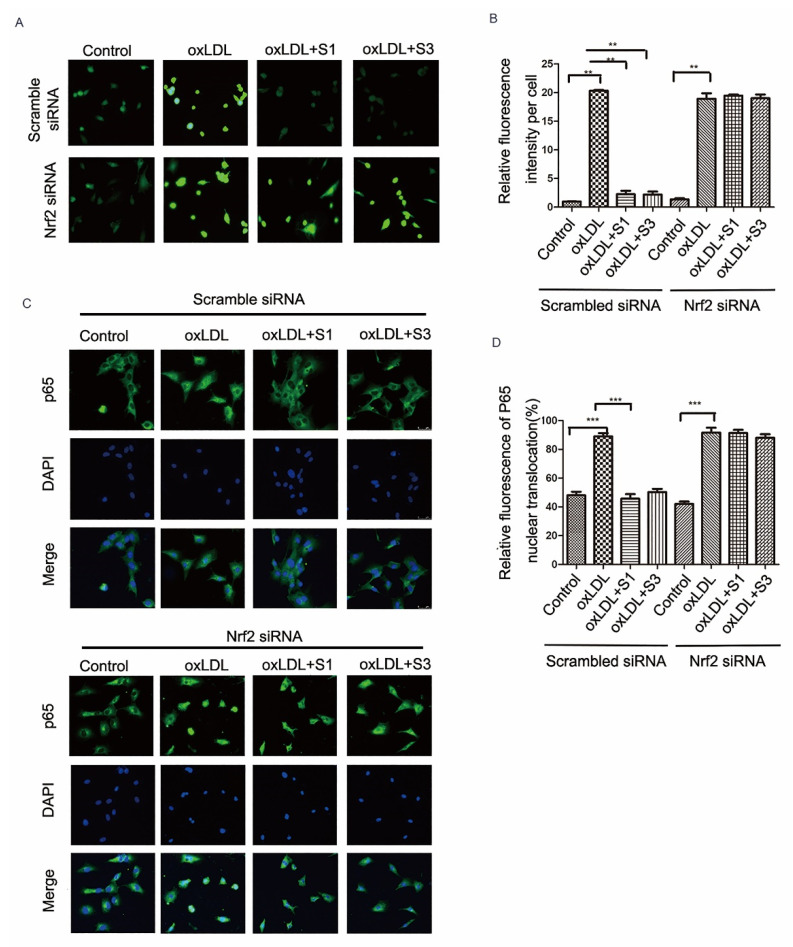
Effects of compounds S1 and S3 on the ROS production and the activation of NF-κB in Nrf2-knockdown HUVECs. HUVECs were treated with 40 nM scramble siRNA or Nrf2 siRNA for 48 h and treated with oxLDL (50 μg/mL) or oxLDL and indicated compound for up to 6 h. (**A**) The ROS level in HUVECs was examined by DCHF (100×). (**C**) Fluorescent micrographs showed the distribution of NF-κB p65 in HUVECs (200×). (**B**,**D**) Quantification of intracellular ROS level and NF-κB p65 nuclear translocation. Data were analyzed using one-way ANOVA with Bonferroni correction. ** *p*< 0.01, *** *p* < 0.001 between indicated groups. *n* = 3.

To further determine whether Nrf2 is directly involved in cell protection of dihydrohomoplantagin and homoplantaginin, cells were transfected with either scramble siRNA or siNrf2 RNA. Hoechst 33258 staining results revealed that compounds could not inhibit oxLDL-induced apoptosis in siNrf2 groups (Figure 8A). Western blot analysis demonstrated that the effects of compound S1 or S3 on the protein level of Bcl2/Bax, ICAM-1, VCAM-1 and HO-1 were abolished by knocking down of Nrf2 (Figure 8B). Moreover, S1 and S3 inhibition of oxLDL-induced ROS and NF-κB nuclear translocation were reversed by Nrf2 siRNA (Figure 9). Therefore, the above experiments suggested the Nrf2 was a key target of dihydrohomoplantagin and homoplantaginin.

## 3. Discussion

**Figure 10:**
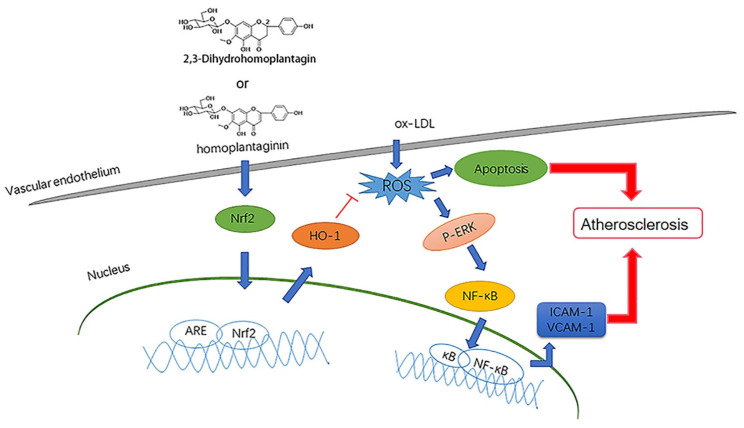
Scheme summarizing the mechanism for inhibition effects of dihydrohomoplantagin (S1) and homoplantaginin (S3) on oxLDL-induced VEC injury and atherosclerosis. OxLDL induces ROS production and NF-kB nuclear translocation by inducing ERK phosphorylation, which contributes to VEC apoptosis, adhesion molecule expression and thus the atherosclerosis development. Dihydrohomoplantagin and homoplantaginin activate Nrf2 nuclear translocation and the target gene HO-1 expression, which suppress cellular ROS accumulation, the phosphorylation of ERK and NF-κB activation induced by oxLDL.

The present study demonstrated the novel role of homoplantaginin and dihydrohomoplantagin in protection against oxLDL-induced injury in endothelial cell and the development of atherosclerosis in apoE-/- mice. Homoplantaginin and dihydrohomoplantagin exhibited the protective effects through activating the Nrf2/HO-1 anti-oxidation signal pathway (Figure 10).

Endothelial cell injury has been confirmed to be the initial step in the atherosclerosis development [15]. It is believed that oxLDL induces endothelium injury and enhances atherosclerosis by increasing endothelial cell oxidative stress [16]. Thus, inhibiting oxLDL-induced endothelial cell injury has becoming a promising strategy to prevent the development of atherosclerosis [4]. Recently, homoplantaginin and dihydrohomoplantagin were isolated from the traditional Chinese herbal medicine *Salvia plebeia* R. BR. (Labiatae) by our research group. Homoplantaginin and dihydrohomoplantagin belong to flavone glucopyranosides compounds. It is known that flavone, such as *Salvia plebeia* extract, hesperetin, naringenin, eriodictyol, isosakuranetin, and their respective glycosides, support and enhance the body’s defenses against oxidative stress and help the organism in the prevention of cardiovascular diseases [10,17]. Moreover, eight flavone compounds including luteolin 4’ methyl ether 7-*O*-β-d-4C1-glucopyranoside 8-methoxyapigenin 7-*O*-β-d-4C1-galactopyranoside, isovitexin, 8-methoxyluteolin 7-*O*-β-d-4C1-glucopyranoside, diosmetin, cirsimaritin, luteolin, and apigenin have been found to exhibit anti-inflammatory, antidiabetic, antioxidant activities, and anti-atherosclerosis in a dose-dependent manner [18]. Previous studies also confirmed that natural flavonoid Vitexin, which was identified as apigenin-8-C-b-d-glucopyranoside, exhibited anti-oxidative and anti-inflammatory properties in PC12 cells [19]. Although homoplantaginin has displayed various biological properties including inhibition of free fatty acids-induced endothelial insulin resistance and endothelial inflammation [9,10,11,12], whether homoplantaginin alleviate vascular endothelial cells injury induced by oxLDL and inhibit atherosclerosis is unclear. Here, we found that homoplantaginin and dihydrohomoplantagin significantly inhibited apoptosis and the increased ICMA-1 and VCAM-1protein level in oxLDL-stimulated HUVECs and endothelium of apoE-/- mice. Importantly, dihydrohomoplantagin and homoplantaginin enhanced atherosclerotic plaque stability of apoE-/- mice.

It is reported that increased ROS production induced by oxLDL promotes NF-κB translocation into the nuclei and thus facilitates vascular endothelial cell injury [20]. Moreover, numerous studies have shown that ROS accumulation can activate ERK/NF-κB pathways [21,22]. Here, the results demonstrated that dihydrohomoplantagin and homo-plantaginin significantly inhibited the increased ROS level in oxLDL-stimulated HUVECs and atherosclerotic lesion of apoE-/- mice, respectively. Moreover, the increased p-ERK level and the NF-κB nuclear translocation induced by oxLDL was significantly inhibited by dihydrohomoplantagin and homoplantaginin, indicating dihydrohomoplantagin and homoplantaginin inhibited ROS/ERK/NF-ĸB signaling pathway.

Nrf2 has been confirmed to be involved in several detoxifying and antioxidant defense processes by inducing anti-oxidative gene expression. As the Nrf2 targeted gene, HO-1 expression was demonstrated to play an important role against oxidative stress [23]. It is reported that 7,8-Dihydroxyflavone activated Nrf2/HO-1 signaling pathways and protected against osteoarthritis [24]. Additionally, methylnis-solin-3-*O*-β-d-glucopyranoside protected VECs against oxidative damage through the Nrf2/HO-1 pathways [25]. Meanwhile, Apigenin-7-*O*-β-d-(-6″-p-coumaroyl)-glucopyranoside treatment elicited a neuro-protective effect through Nrf2 activation [26]. However, whether dihydrohomoplantagin and homoplantaginin exert antioxidant effect by activating Nrf2 remains unclear. Here, we found that dihydrohomoplantagin and homoplantaginin promoted Nrf2 nuclear translocation and increased the anti-oxidation downstream HO-1 protein level in HUVECs and plaque endothelium. Notably, the down-regulation of Nrf2 by small siRNA of Nrf2 abolished the cell protective effects of compounds and antagonized the inhibition effects of them on ROS production and NF-κB activation in oxLDL-stimulated HUVECs. These results suggested dihydrohomoplantagin and homoplantaginin protected VECs against oxLDL-induced oxidative injury by activating Nrf2.

In summary, our results revealed that dihydrohomoplantagin and homoplantaginin significantly inhibited oxLDL-induced VEC apoptosis and the expression of ICAM-1 and VCAM-1. The research elucidated that dihydrohomoplantagin and homoplantaginin inhibited oxLDL-induced ROS overproduction, the phosphorylation of ERK and NF-κB activation through activating Nrf2/HO-1 anti-oxidative signaling pathway. Collectively, dihydrohomoplantagin and homoplantaginin protected VECs by activating Nrf2 and thus restricted atherosclerosis in apoE-/- mice. Therefore, our study provides a lead compound for the treatment of atherosclerosis.

## 4. Materials and Methods

### 4.1. Preparation of Dihydrohomoplantagin and Homoplantaginin

The dry herbs of *Salvia plebeia* R. BR. were purchased from Qilu hospital (Jinan, China), then powdered, refluxed with 60% ethanol two times and 95% ethanol two times. The evaporated extract was suspended in water and successively partitioned with petroleum ether and n-butanol. The ethyl acetate fraction was subjected to polyamide column chromatography (60~80 mesh) eluted by with a gradient of CH_3_OH/H_2_O (1:5→100:0). Dihydrohomoplantagin was obtained from the 20% CH_3_OH elution using silica gel column chromatography CH_2_Cl_2_/H_2_O (9:1) and high-performance liquid chromatography (HPLC), homoplantaginin was obtained from the 30% CH_3_OH elution using silica gel column chromatography, then eluted by CH_2_Cl_2_/H2O (10:1 and 100:13) and HPLC. Two chemical structures were identified through spectroscopic analysis as reported previously [7,27].

### 4.2. Cell Culture and Treatment

HUVECs were obtained from ScienCell (San Diego, CA, USA) and cultured on gelatincoated plastic dishes in M199 medium with 20% (*v*/*v*) bovine serum and 10 IU/mL fibroblast growth factor 2 (FGF2). The cells were maintained at 37 °C under humidified conditions and 5% CO2. HUVECs at no more than passage 25 were used in the experiments. When HUVECs were grown to 80% confluency, the cells were stimulated with oxLDL or oxLDL and compounds.

### 4.3. Antibodies

The primary antibodies used for Western blot and immunofluorescence staining including Bcl-2, Bax, HO-1, ICAM-1, VCAM-1, phospho-ERK, and ERK were purchased from Proteintech Group, Inc. (Chicago, IL, USA). The corresponding peroxidase conjugated secondary antibodies used for Western blot were acquired from Dingguo Changsheng Biotechnology Co., Ltd. (Beijing, China).

### 4.4. Western Blot Analysis

Protein was isolated from HUVECs using radio immune precipitation assay (RIPA) lysis buffer (Dingguo Changsheng Biotechnology Co., Ltd., Beijing, China). The total protein concentration was determined using a quantitative BCA protein kit (Beyotime, Shenzhen, Guangdong, China). Proteins from different groups were mixed with 5× gel loading buffer and boiled for 5 min. Equal amounts of protein (20 μg) were separated by SDS-PAGE. Separated proteins were transferred to PVDF membranes and then the membranes were incubated in 5% non-fat milk for 1 h at 37 °C to block the non-specific binding. After that, incubation with specific primary antibodies (1:500) for targeted proteins was performed for 18 h at 4 °C. After three washes in Tris-buffered saline/Tween 20 for 10 min for each time, membranes were incubated with corresponding HRP-conjugated secondary antibody (1:2000) for 1.5 h at room temperature. The protein bands were detected by enhanced chemiluminescence (Millipore Corporation, Billerica, MA, USA). The β-actin was used as the internal control.

### 4.5. Hoechst 33258 Staining

Nuclear fragmentation of apoptotic cells was determined by Hoechst 33258 staining. After treatment, HUVECs were incubated with Hoechst 33258 solution (10 mg/mL, Dingguo Changsheng Biotechnology Co., Ltd., DH163) for 20 min at 37 °C, and then were detected using a fluorescence microscopy (LEICA DMi8, Wiesbaden, Germany).

### 4.6. Intracellular ROS Assay

HUVECs were incubated with the carboxy-2′,7′-dichlorodihydrofluorescein (DCHF, Invitrogen, Carlsbad, CA, USA) probe for 30 min. After incubation, samples were washed with medium M199 3 times and then the cellular fluorescence which reflected the intracellular ROS level was detected using a fluorescence microscopy at the 488 nm examination wavelength.

### 4.7. Cells for Immunofluorescence Staining

After treatment, HUVECs were fixed in 4% paraformaldehyde (*w*/*v*) for 15 min at room temperature and blocked with normal donkey serum (Solarbio, Beijing, China) for 30 min, and incubated with anti-Nrf2 or NF-κB antibody for 12 h at 4 °C. Then, the samples were washed with PBST (0.05% Tween-20 in PBS) 3 times and were incubated with corresponding fluorescence labeled secondary antibody (1:200). After washing with PBS, the cellular immunofluorescence was evaluated by a fluorescent microscope.

### 4.8. TUNEL Staining

A terminal deoxynucleotidyl transferase-mediated biotinylated UTP nick end labeling (TUNEL) assay was performed according to the manufacturer’s instructions (Roche, Switzerland, France). Briefly, the HUVECs were fixed with 4% paraformaldehyde for 30 min and subsequently permeabilized with 0.25% Triton X-100 in 0.1% sodium citrate for 5 min at room temperature. Finally, the HUVECs were incubated with TUNEL Enzyme-TMR label mixture solution. The TUNEL-positive HUVECs were monitored by a fluorescence microscope.

### 4.9. Animal Model

6 weeks of male apoE-/-mice on a C57BL/6J background were purchased from the Department of Laboratory Animal Science, Peking University Health Science Center (Beijing, China). ApoE-/- mice were fed an atherogenic diet (containing 21% fat and 0.15% cholesterol) for 12 weeks. Then, the mice were randomly divided into five groups (n = 8). The mice of each different group were intravenously injected with saline solution (control), S1 (5 mg/kg), S1 (10 mg/kg), S3 (5 mg/kg) or S3 (10 mg/kg) for six weeks, respectively. Mice were sacrificed after injection and the aortic roots were dissected and embedded in optimal cutting temperature (OCT) embedding medium (Tissue-Tek, Sakura Finetek USA, Torrance, CA) for histology and immunofluorescence assay.

All animal experiments complied with the ARRIVE guidelines and were carried out in accordance with the U.K. Animals (Scientific Procedures) Act, 1986 and associated guidelines, EU Directive 2010/63/EU for animal experiments, and were approved by the ethics committee for laboratory animals of University of Jinan.

### 4.10. Histological and Immunofluorescence

Oil red O (Sigma-Aldrich, Zwijndrecht, The Netherlands) staining was used to assess atherosclerotic plaque area and lipid accumulation in the aorta or aortic root following the manufacturer’s protocol. Image-Pro Plus 6.0 software (Media Cybernetics, CA, United States) was applied to analyze the positive staining within the aortas. The red or orange areas were considered positive regions. The area of an aortic atherosclerosis lesion was represented by the percentage of the oil red O positively stained area relative to the whole aortic intima area.

Immunofluorescence staining was performed to detect the target protein levels in endothelium of plaque. The sections (7 μm thick) were fixed in cold acetone for 10 min, blocked with 5% BSA containing 0.2% Triton X-100 for 2 min, after which they were incubated with primary antibody (1:200) overnight at 4 °C. After incubation with the appropriate secondary antibodies (1:500) at 37 °C for 1.5 h, sections were observed by confocal laser scanning microscopy (Zeiss LSM800, Carl Zeiss, Jena, Germany).

H&E staining and Masson staining were performed according to the instructions provided by the manufacturer (Solarbio Biotechnology, Beijing, China). The microscopic images of lesions in the aortic sinus were captured by microscope. Image-Pro Plus 6.0 was used to measure the percentage of lesion area and collagen area.

### 4.11. Statistical Analysis

All the quantifications are expressed as mean ± SEM. Images were processed by the use of Graphpad Prism 5 (GraphPad Software, La Jolla, CA, USA) and Adobe Photoshop (Adobe, San Jose, CA, USA). In vitro experiments were routinely repeated with at least 3 independent biological replicates. Statistical evaluations were performed with the Student’s *t*-test or one-way ANOVA with Bonferroni correction for multiple comparisons. *p* ≤ 0.05 was considered statistically significant.

## Data Availability

The data presented in this study are publicly available.

## Supplementary Materials

The following supporting information are available online https://www.mdpi.com/article/10.3390/molecules27061990/s1, Figure S1: Effect of Compounds on the weight of body; Figure S2: The vulnerability index of plaque in apoE-/- mice with or without compound S1 or S3. Vulnerable index = (the percentage of macrophage positive area + the percentage of lipid positive area) / (the percentage of smooth muscle cell positive area + the percentage of collagen positive area) Data were analyzed by an unpaired two-sided Student’s *t*-test. ### *p* < 0.001, vs. control, n = 6 mice per group; Table S1: Measurement of organ coefficients of mice after treatment with Compounds. The organ coefficient was obtained by dividing the weight of the organ by body weight. n = 6 mice per group.

## Sample Availability

Samples of the compounds are not available from the authors.
