# Supplementary material for: Dihydrohomoplantagin and Homoplantaginin, Major Flavonoid Glycosides from Salvia plebeia R. Br. Inhibit oxLDL-Induced Endothelial Cell Injury and Restrict Atherosclerosis via Activating Nrf2 Anti-Oxidation Signal Pathway
Source: Molecules. 2022 Mar 19;27(6):1990. doi: 10.3390/molecules27061990 (PMC8951125; doi:10.3390/molecules27061990)
Supplement: Supplementary file 1 [file molecules-27-01990-s001.zip › molecules-1617001-supplementary.pdf]

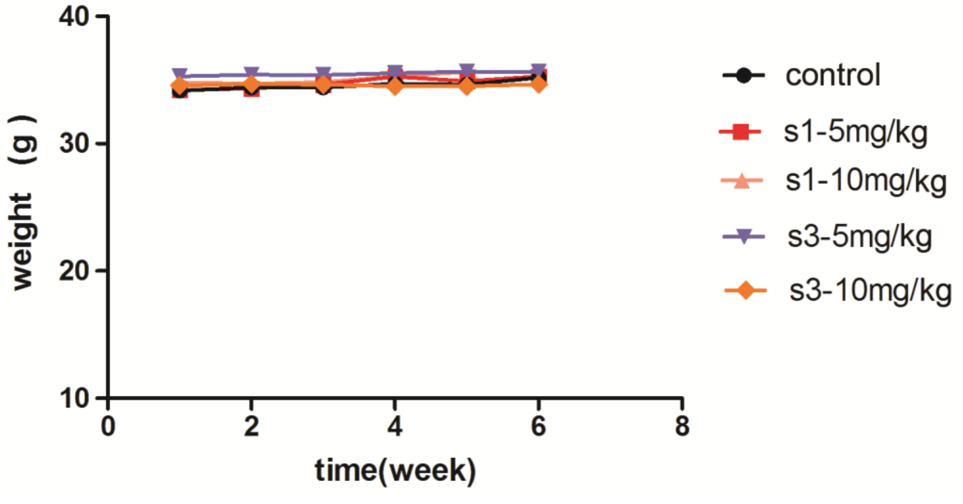

Supplemental Figure S1. Effect of Compounds on the weight of body.

Table S1. Measurement of organ coefficients of mice after treatment with Compounds. The organ coefficient was obtained by dividing the weight of the organ by body weight. n = 6 mice per group.

Groups	Heart (%)	Liver (%)	Spleen (%)	Lung (%)	Kidney (%)
Control	0.42±0.01	5.14±0.45	0.57±0.02	0.55±0.04	1.21±0.10
S1-5mg/kg	0.43±0.03	5.19±0.53	0.56±0.01	0.55±0.03	1.20±0.12
S1-10mg/kg	0.41±0.01	5.15±0.61	0.57±0.02	0.53±0.05	1.21±0.15
S3-5mg/kg	0.44±0.05	5.18±0.66	0.56±0.04	0.52±0.04	1.19±0.16
S3-10mg/kg	0.44±0.03	5.18±0.33	0.57±0.04	0.53±0.04	1.22±0.14

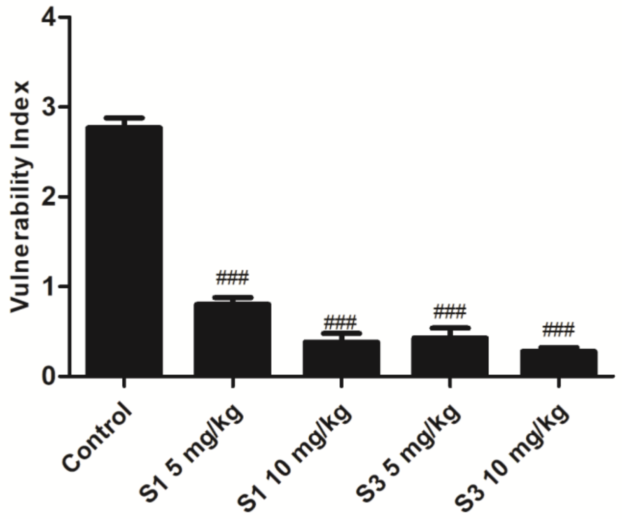

Supplemental Figure S2. The vulnerability index of plaque in apoE$^{-/-}$ mice with or without compound S1 or S3. Vulnerable index = (the percentage of macrophage

positive area + the percentage of lipid positive area) / (the percentage of smooth muscle cell positive area + the percentage of collagen positive area) Data were analyzed by an unpaired two-sided Student's t-test. ###$p < 0.001$, vs. control, n = 6 mice per group.
